# Resilience and Coping With COVID-19: The COPERS Study

**DOI:** 10.3389/ijph.2021.1604007

**Published:** 2021-04-26

**Authors:** Insa Backhaus, Felix Sisenop, Edvaldo Begotaraj, John Cachia, Stefano Capolongo, Mauro G. Carta, Marija Jakubauskiene, Marija Jevtic, Vladimir Nakov, Mihail Cristian Pirlog, Danijela S. Grbic, Matej Vinko, Milica P. Kusturica, Alessandro Morganti, Jutta Lindert

**Affiliations:** ^1^ Institute of Medical Sociology, Centre for Health and Society, Medical Faculty, University of Düsseldorf, Düsseldorf, Germany; ^2^ Department of Health and Social Work, University of Applied Sciences Emden/Leer, Emden, Germany; ^3^ Department of Dynamic and Clinical Psychology, Sapienza University of Rome, Rome, Italy; ^4^ College University “LOGOS”, Tirana, Albania; ^5^ Commissioner for Mental Health, Office of the Commissioner for Mental Health, Msida, Malta; ^6^ Department of Architecture, Built Environment and Construction Engineering (DABC), Design and Health Lab, Politecnico di Milano, Milan, Italy; ^7^ Department of Applied Medical Technologies and Methodology, University of Cagliari, Cagliari, Italy; ^8^ Faculty of Medicine, Vilnius University, Vilnius, Lithuania; ^9^ Faculty of Medicine, University of Novi Sad, Novi Sad, Serbia; ^10^ Institute of Public Health of Vojvodinia, Novi Sad, Serbia; ^11^ Department of Mental Health, National Center of Public Health and Analyses, Sofia, Bulgaria; ^12^ Faculty of Medicine, University of Medicine and Pharmacy of Craiova, Craiova, Romania; ^13^ Faculty of Medicine, Zagreb University, Zagreb, Croatia; ^14^ National Institute of Public Health, Ljubljana, Slovenia; ^15^ WRSC, Brandeis University, Waltham, MA, United States

**Keywords:** COVID-19, mental health, resilience, Europe, longitudinal study

A year has passed, and the COVID-19 pandemic continues to spread around the world. Apart from its direct devastating health consequences, voices have been raised about decreasing mental health. [1] The introduction of social distancing measures and lockdown to prevent the spread of COVID-19 led to a sudden change of routine, a drastic change of the physical and social environment (e.g., reduced social contacts and connection), a change of working conditions and economic losses, all are known to be important drivers for mental health and mental disorders.

Evidence from previous studies that have examined the psychological impact of disaster and outbreaks, shows that such health emergencies have its toll on mental health. In Hong-Kong, for example, suicide rates rose sharply during the 2003 SARS epidemic [2].

Given that measures taken during the SARS epidemic interfered much less with normal life and for a shorter period, it is not far-fetched to assume that the COVID-19 pandemic, in one way or another, will affect mental health. In fact, surveys conducted so far, using contemporary data from national surveys, show that since the start of the pandemic, mental health has deteriorated [2, 3]. Current research suggests that poorer mental health is associated with increased fear of illness, social distancing policies and social adversities (e.g. loss of employment), housing conditions as well as loss of support from health and other services [4, 5]. As a consequence of the lockdown and social distancing measures, psychosocial services as well as in- and out-patient mental health care facilities greatly interrupted, reduced and changed their services [4]. Many services switched from face-to-face to digital services and check-in phone calls [4]. However, due to lack of access or ability to use technology and a lack of privacy, remote care is not always considered sufficient [4]. Additionally, people at risk, might feel stigmatized due to the perceptions of being a burden to society. The epidemic may shatter personal goals and way of living and undermine a sense of meaning in life and trust in others. Meaning making, however, is a predictor of resilience and mental health.

Resilience is a multidimensional concept which can be defined as being able to adapt to stressful (e.g., family and relationship problems, workplace and financial stressors) and extraordinary threatening events, such as a pandemic. The factors, which contribute to resilience, are manifold, including individual, interpersonal and community factors [1]. At the individual level, very recent research suggests that healthcare personnel, youth and older individuals, and those with a current or past medical history, especially a history of mental illness, are at risk [1, 3]. Additionally, we learnt from other disasters that at the interpersonal level, social isolation, and lack of trusting relationships and at the society level lack of trust in the government might put people at risk.

While evidence about the effects of COVID-19 on resilience and mental health is emerging, at this point, no accurate assumptions about the extent of the pandemic on mental health can be made. Furthermore, the mental health responses to the pandemic and the trajectories of mental health and resilience remain unknown. Approaches to mental health and psychiatric care in such outbreaks remain poorly understood, outlined or covered by existing research and there is virtually no substantial knowledge of the mental health impact of rapidly spreading outbreaks of infectious diseases. International cooperation is essential for building appropriate mental health prevention and promotion strategies. Identifying factors that may contribute to better mental health and greater psychological resilience at an international level and sharing experiences and information is of utmost importance. Therefore, a comprehensive assessment of the prevalence, risk and protective factors, of mental health is necessary.

For that reasons the international Public Mental Health Section of the European Public Health Association (EUPHA) initiated this study and worked together with the consortium members to set up the Coping with COVID-19 with Resilience (COPERS) study. The consortium includes members of 10 countries all over Europe. COPERS aims to grasp longitudinally the extent of mental health and resilience across Europe, to identify mental health, and resilience trajectories of adults aged 18 years and older and to detect factors that potentially influence mental health and resilience in the different countries. The researchers also aim to conduct a living mapping review to identify, analyze, and present mental health policy responses to the COVID-19 pandemic and contribute to better knowledge on how to support personal and community resilience in times of a pandemic.
